# Comment: Trends in Cigarette Smoking Among United States Adolescents

**DOI:** 10.31486/toj.23.0145

**Published:** 2024

**Authors:** Muhammad Hasham Khawaja, Omna Daulat Khawaja

**Affiliations:** ^1^ Department of Internal Medicine, Nishtar Medical University and Hospital, Multan, Punjab, Pakistan; ^2^ Department of General Surgery, Bakhtawar Amin Medical and Dental College and Hospital, Multan, Punjab, Pakistan

## TO THE EDITOR

We write this letter in response to an article published in *Ochsner Journal* titled “Trends in Cigarette Smoking Among United States Adolescents” that describes the decreasing trend of cigarette smoking among high school–aged youth.^1^ While Mejia et al shed light on very encouraging statistics of decreased use of cigarettes among adolescents, we would like to mention the increased propensity of the younger generation to use electronic cigarettes, also called e-cigarettes. According to the “Tobacco Product Use Among U.S. Middle and High School Students National Youth Tobacco Survey, 2023” from the Centers for Disease Control and Prevention (CDC), among young persons who had ever tried e-cigarettes, almost half reported current use,^2^ posing a risk of dependence. A study highlighting trends of tobacco and nicotine product use needs to be conducted among children in middle school (grades 6 to 8), as the CDC survey showed an overall increase in the use of any tobacco product or multiple tobacco products among that age group from 2022 to 2023.^2^

E-cigarettes are considered to be a safer alternative to cigarettes,^3^ but e-cigarettes have adverse effects, including damage to the lungs.^3^ Consequently, there is a dire need to create awareness among young people regarding these ill effects. Social media can be used to engage target audiences. In terms of awareness campaigns, the US Preventive Services Task Force recommendations suggest that behavioral counseling and education are helpful in preventing initiation among school-aged children and adolescents who have not yet started smoking.^4^ In addition, regulations on the pricing, distribution, and sale to young people would help control the use of e-cigarettes.
